# The role of sp^2^ and sp^3^ hybridized bonds on the structural, mechanical, and electronic properties in a hard BN framework

**DOI:** 10.1039/c8ra09636h

**Published:** 2019-01-21

**Authors:** Hongxia Bu, Haibin Zheng, Hongcai Zhou, Hongyu Zhang, Zaifa Yang, Zhie Liu, Hui Wang, Qi Xu

**Affiliations:** College of Physics and Electronic Engineering, Qilu Normal University Jinan Shandong 250200 China buhx666@163.com haibin_zheng@126.com +86-531-66778300; Science and Information College, Qingdao Agricultural University Qingdao Shandong 266109 China; Department of Physics, East China University of Science and Technology Shanghai 200237 China

## Abstract

A first-principles approach is used to systematically investigate the role of sp^2^ and sp^3^ hybridized bonds on the structural, mechanical, and electronic properties in a new BN phase (denoted Hex-(BN)_12_). Hex-(BN)_12_ has the same number of sp^2^ and sp^3^ hybridized atoms. The calculated cohesion energy, phonon frequencies, and elastic constants unambiguously confirm the structural stability of this compound. Due to the different types of hybridization and B–N covalent bonds with ionic characteristics, Hex-(BN)_12_ has unequal bond lengths and bond angles in these hybrid orbitals. These cause the relative energetic stability to be slightly lower than c-BN and w-BN. The hardness of Hex-(BN)_12_ is estimated to range from 33 to 40 GPa. The bond-breaking order under stress is sp^3^–sp^3^, sp^2^–sp^3^, and sp^2^–sp^2^. DFT calculations with the gradient approximation (GGA) and HSE06 functional indicate the electronic structure contains an indirect band gap at 3.21 and 4.42 eV, respectively. The electronic states in the region near the Fermi level primarily arise from the 2p orbitals in sp^2^-hybridized atoms. In general, sp^3^ bonded B and N atoms guarantee higher mechanical properties, and sp^2^ bonded atoms ensure ductility and even conductivity, although all changes vary with spatial structure. Hex-(BN)_12_ can be obtained from multilayer yne-BN, and BN nanosheets, nanotubes and nanoribbons under pressure.

## Abstract 2

A first-principles approach is used to systematically investigate the role of sp^2^ and sp^3^ hybridized bonds on the structural, mechanical, and electronic properties in a new BN phase (denoted Hex-(BN)_12_).

## Graphical abstract

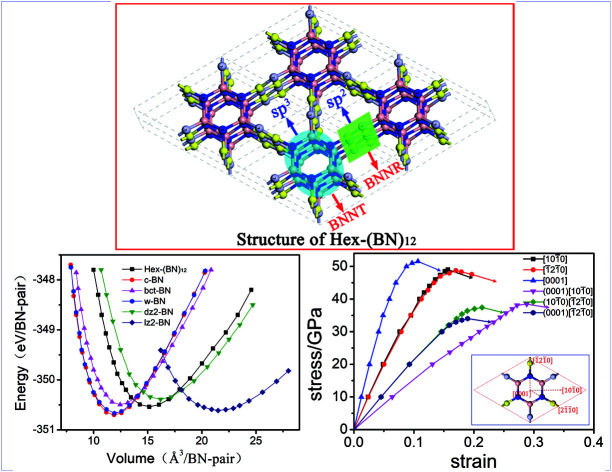

## Introduction

Boron nitride (BN) is a group III–V compound that can theoretically exhibit a variety of frameworks because B and N atoms can form chemical bonds by means of sp, sp^2^, and sp^3^ hybridizations or combinations. It is an intriguing compound that has been studied for many years, and numerous BN structures were theoretically proposed or experimentally synthesized in the past few decades. Examples include sp and sp^2^ graphyne-BN (yne-BN) and graphdiyne-BN (diyne-BN);^1–5^ sp^2^-hybridized nanocages,^6–8^ nanotubes,^9–12^ nanoribbons^13,14^ and nanosheets;^15–18^ sp^2^ and sp^3^ porous BN networks,^19^ M-BN,^20^ HBNFs,^21^ and 3D BN allotropes from compressed single-walled BN nanotubes (BNNTs);^22^ sp^3^-hybridized c-BN, bct-BN, Z-BN,^23^ and O-BN.^24^ They usually have high thermal conductivity, chemical stability, excellent mechanical properties, and unique electronic and optical properties, facilitating practical applications in the many fields related to hydrogen energy,^19,25,26^ advanced abrasives,^27^ ultraviolet laser devices,^28,29^ nanoscale spintronic devices,^30^ nanomedicine,^31,32^ and flexible resistive memory devices.^33^

Meanwhile, it is worth noting these BN phases exhibit unique physical and chemical properties. For example, they form many crystal structures, ranging from zero to three dimensional. The mechanical properties of these reported BN phases are different. c-BN and w-BN are typical superhard materials; M-BN is hard, while h-BN formed from sp^2^-hybridized atoms is more ductile and is widely used as a lubricant. Furthermore, given the same fundamental sp^2^ and sp^3^ hybridized bonds, their electronic structures are also different. The metallicity of T-B_3_N_3_ originates from sp^2^ hybridized B atoms,^34^ while M-BN is metallic with one-dimensional metallicity from sp^2^ B/N atoms.^20^ However, dz2-BN, lz2-BN, 3D(*n*,0)-I (*n* = 6, 8, 10, 12), and Na-HBNFs are semiconductors.^19,21,22^ All these characteristics indicate that the properties of BN are highly dependent on the crystalline structure, including the type of hybridization. However, fewer studies have focused on the influence from sp^2^ and sp^3^ hybridized bonds in 3D multiporous BN polymorphs. Moreover, the influence of sp^2^ and sp^3^ hybridization on stability, hardness, and electronic properties is an interesting issue that is worth studying.

In this work, we present a 3D hard BN skeleton consisting of both sp^2^ and sp^3^ hybridized bonds. This BN phase has hexagonal structure with 12 pairs of BN in a conventional unit cell. Thus, we have denoted this BN phase as Hex-(BN)_12_. The structure of Hex-(BN)_12_ is composed of narrow BN nanoribbons (BNNRs) interconnected by ultrathin BNNTs. Hex-(BN)_12_ is energetically, dynamically, and mechanically stable, and it is energetically more favorable than yne-BN. Calculations with the gradient approximation (GGA) and HSE06 functional show the presence of an indirect band gap of 3.21 and 4.42 eV at the Brillouin zone in Hex-(BN)_12_, respectively. We conducted a systematic first-principles study on the effect of sp^2^ and sp^3^ hybridizations on the structural, mechanical, and electronic properties of Hex-(BN)_12_.

### Computational methods

First-principles calculations were performed using the CASTEP code, which is based on density functional theory (DFT).^35,36^ Electron–electron interactions were modeled using a generalized gradient approximation (GGA) according to Perdew, Burke, and Ernzerhof.^37^ The cutoff energy was set to 400 eV for the plane-wave basis within an ultrasoft pseudopotential.^38^ The *k*-point separation for Brillouin zone sampling was set to 0.02 Å^−1^ in accordance with the Monkhorst–Pack method.^39^ Lattice constants and internal coordinates were optimized within the Broyden–Fletcher–Goldfarb–Shanno (BFGS) minimization scheme.^40^ Phonon dispersion spectra were calculated using linear response and finite displacement theories in the CASTEP code. The standard GGA functional always underestimates the band gap in semiconductors, thus the band structure was calculated with a more accurate HSE06 hybrid functional^41^ as implemented in the CASTEP code within a norm-conserving pseudopotential.^42^ The convergence plane wave cutoff energy was set to 700 eV.

The elastic constants (*C*_ij_) were theoretically calculated using Hooke's law (*σ*_i_ = *C*_ij_*ε*_j_) for small stresses (*σ*) and strains (*ε*).^43^ Young's, bulk, and shear moduli were estimated using the Voigt–Reuss–Hill approximation.^44^ Vickers hardness was calculated using Chen's^45^ and Gao's^46^ hardness models.

On the basis of the equilibrium structure, a series of incremental stress were applied to the structure in order to determine its structural response to stress. At each step, a desired target stress component was defined along particular directions. The final structure and the corresponding strain were defined as lattice vectors and the atomic positions were fully relaxed simultaneously. In this way, the stress and strain relationship for tensile and shear deformation and the ideal strength were obtained as the structure collapsed.

## Results and discussion

**Fig. 1:**
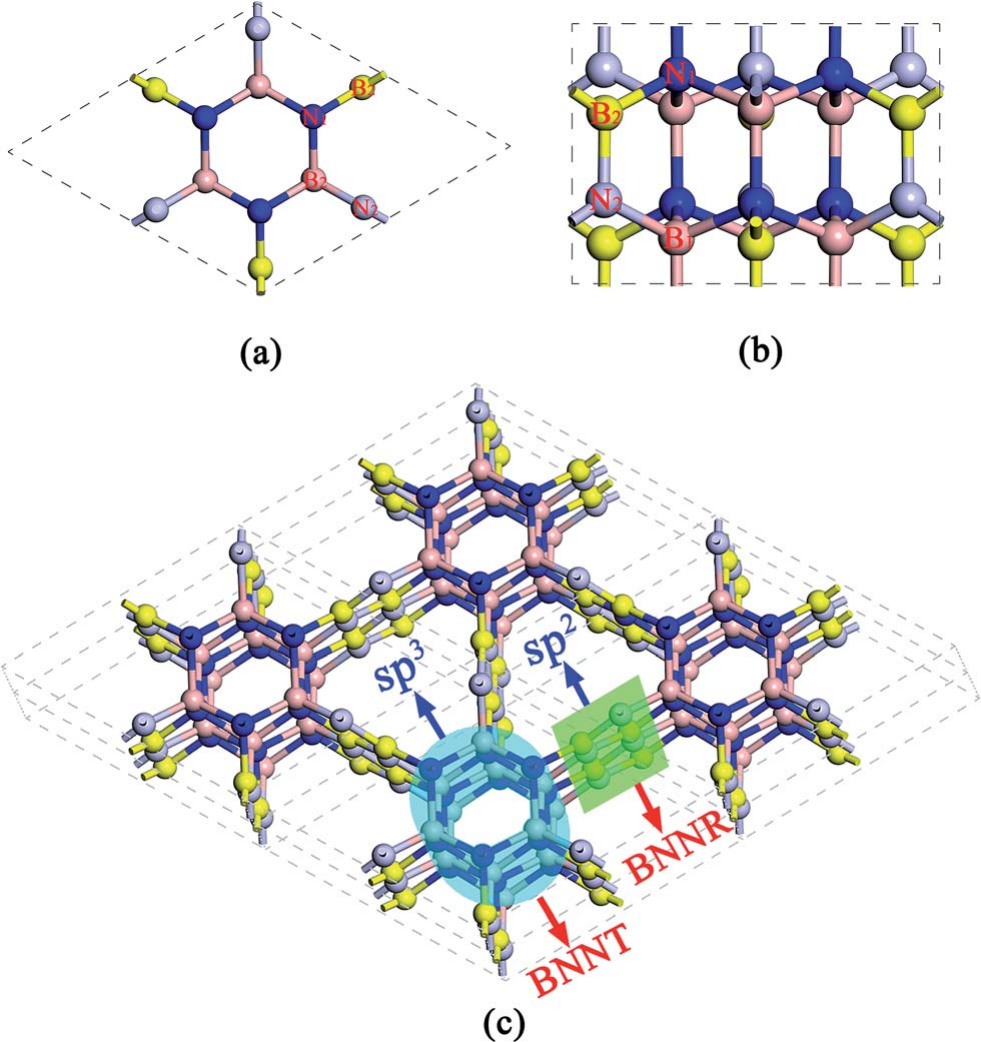
Atomic structure of Hex-(BN)_12_ containing sp^2^- and sp^3^-hybridized B and N atoms. (a) Top view, (b) side view, and (c) schematic representation of the superlattice formed from BNNRs (sp^2^ hybridization) and BNNTs (sp^3^ hybridization).

The predicted crystal structure is shown in Fig. 1 from different views. B and N atoms in the basal plane alternately bind to two adjacent layers through the polar B–N covalent bond. The structure has hexagonal symmetry with *P*6_3_*cm* space group (*C*_6V_^3^, no. 185). It contains 12 BN pairs per primitive cell, thus the compound is referred to as Hex-(BN)_12_ hereafter. The fully relaxed lattice constants are *a* = *b* = 6.995 Å and *c* = 4.288 Å. Two inequivalent B atoms and two inequivalent N atoms are present in each primitive cell. B atoms occupy the 6*c* (0, 0.588, 1.143), 6*c* (0.216, 0, 1.164) Wyckoff positions, while N atoms occupy the 6*c* (0, 0.214, 0.793), 6*c* (0.586, 0, 0.811) Wyckoff positions. The framework of Hex-(BN)_12_ is composed of sp^2^- and sp^3^-hybridized B and N atoms, thus it can be thought of as a superlattice of ultrathin BNNTs and narrow BNNRs, as shown in Fig. 1c. The sp^3^-hybridized B and N atoms form ultrathin BNNTs, while sp^2^-hybridized atoms form narrow BNNRs. Notably, the BNNT in this framework differs significantly from the isolated BNNT because B and N atoms in the former are sp^3^-hybridized.

The density of Hex-(BN)_12_ is 2.72 g cm^−3^ as the ratio of sp^2^ BN pairs to sp^3^ BN pairs is 6/6. The density was theoretically calculated using *ρ* = *m*/*V*, where *ρ* is the density, and *m* and *V* are the mass and volume of the primitive cell, respectively. This density value lies between the values for pure sp^2^-hybridized h-BN (2.27 g cm^−3^) and pure sp^3^-hybridized c-BN (3.47 g cm^−3^).

**Fig. 2:**
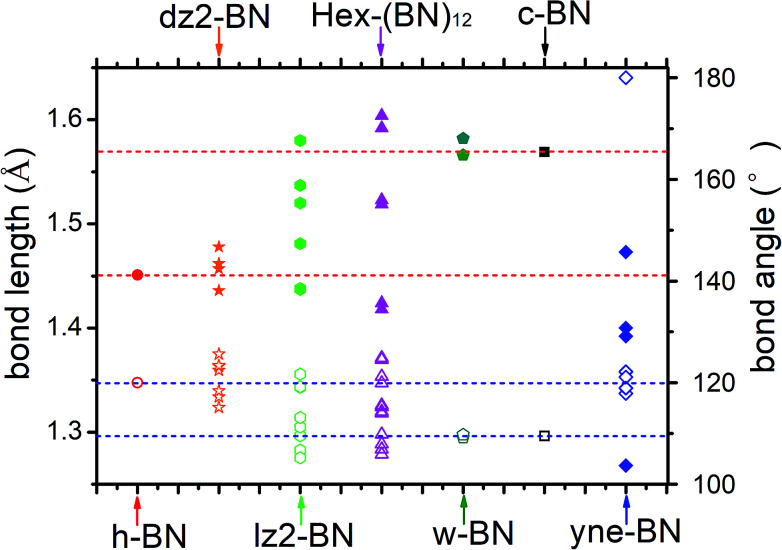
Bond lengths and bond angles in h-BN, dz2-BN, lz2-BN, Hex-(BN)_12_, w-BN, c-BN, and yne-BN. The solid and hollow symbols represent bond lengths and bond angles, respectively. The red and blue horizontal dashed lines indicate bond lengths and angles in h-BN and c-BN.

The optimized bond lengths and angles are shown in Fig. 2. There exists six distinct bonds and twelve bond angles. The lengths of two distinct sp^2^–sp^2^ bonds in the BNNR component are 1.418 and 1.424 Å; these values are slightly shorter than in h-BN (1.451 Å) and in a pristine BN sheet (1.452 Å).^47^ The lengths of the sp^3^–sp^3^ bonds in the BNNT component are 1.592 and 1.604 Å, which is slightly longer than the calculated value for c-BN's (1.569 Å). The lengths of the sp^2^–sp^3^ bonds connecting BNNTs and BNNRs are 1.519 and 1.523 Å. The bond angles deviate slightly from the corresponding standard values in c-BN (109.5°) and in an h-BN monolayer (120°). For example, ∠B–N–B and ∠N–B–N in the sp^2^–sp^2^ bonds in BNNRs have slightly different angles of 119.91° and 121.1° (listed in Fig. 2), respectively, which differs slightly from the angle in an h-BN monolayer (120°). The same applies to other bond angels located at different positions. The different bond patterns and angles can be attributed to two possible explanations. First, different hybrid orbitals of B and N atoms have an important influence on the bond lengths and angles. Second, although B–N bonds in this phase are covalent in nature, they have ionic characteristics. The ionic characteristics have little influence on the bond lengths and angles. We think the significant differences in bond lengths and angles result from the former explanation, while small differences result from the latter. Moreover, Mulliken analysis further verifies the polarity of these B–N bonds, and the results are reasonably consistent with results published in the literature.^1^

**Fig. 3:**
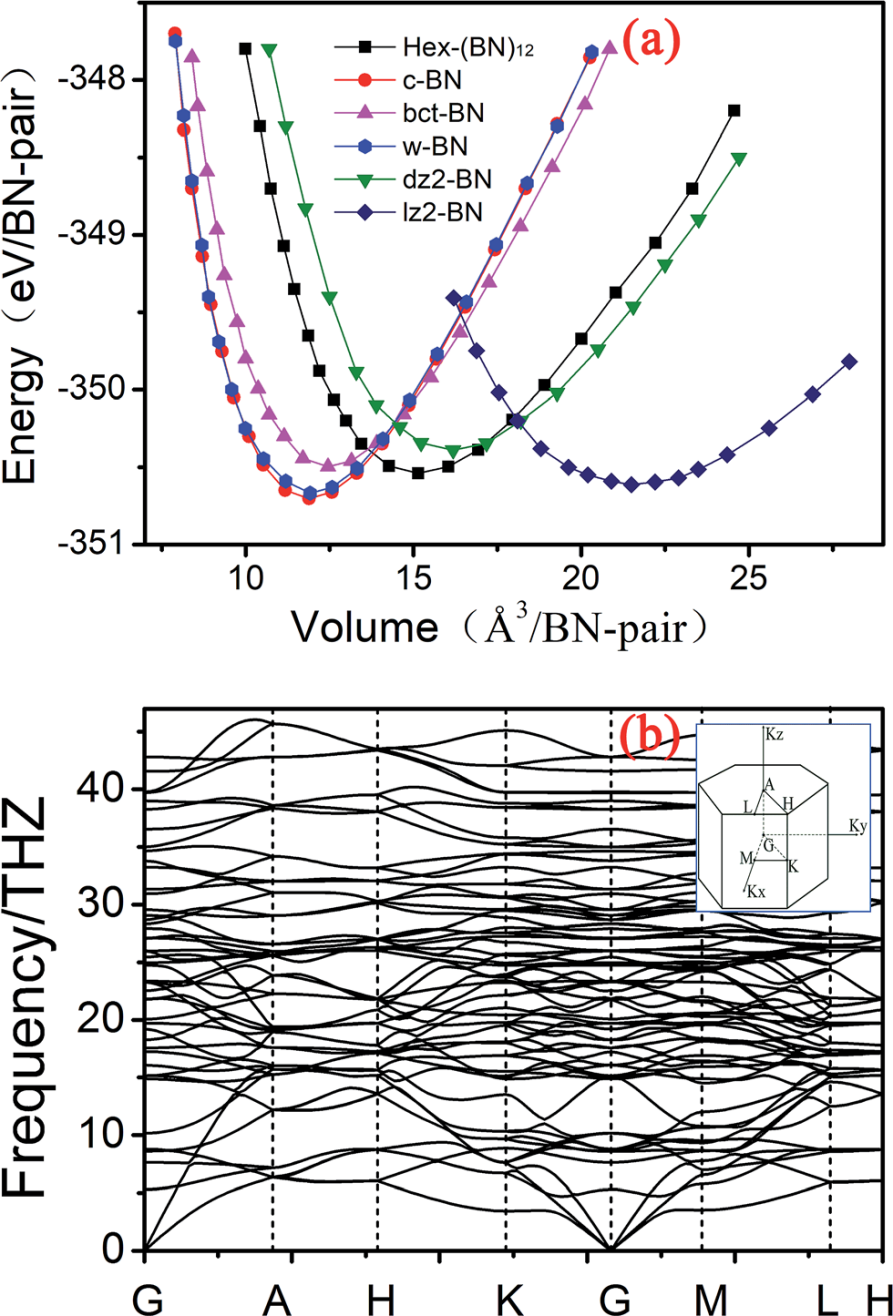
(a) Total energy as a function of volume per BN pair in Hex-(BN)_12_ compared with previously reported BN phases. (b) Phonon band structure in Hex-(BN)_12_.

To evaluate the relative stability of the predicted porous BN phase, we calculated the cohesive energy and compared it with several known BN phases. The cohesive energy is defined by the following equation:*E*_coh_ = (*nE*_B_ + *nE*_N_ − *E*_(BN)*n*_)/*n*where *E*_B_, *E*_N_, and *E*_(BN)*n*_ are the total energies of a single B atom, N atom, and (BN)_*n*_ molecule, respectively. The cohesive energy is comparable to those of bct-BN, lz2-BN, even c-BN, w-BN, and h-BN at zero pressure. This cohesive energy value is more favorable than the value for yne-BN by about 0.76 eV per BN pair (Table 1 and Fig. 3a). From the cohesive energy of c-BN, h-BN, and yne-BN, sp^2^-hybridization is the energetically most favorable hybridization between B and N atoms, followed by sp^3^-hybridization and sp-hybridization. Compared with sp and sp^2^-hybridized yne-BN, Hex-(BN)_12_ contains sp^3^-hybridized instead of sp-hybridized BN pairs, and the number of sp^2^ and sp^3^ hybrids is equal. Therefore, it is not surprising that Hex-(BN)_12_ is energetically favored over yne-BN, and its cohesive energy is comparable even to those of c-BN and w-BN. Furthermore, the unequal bond lengths and bond angles in these hybrid orbitals (as mentioned in the previous paragraph) imply Hex-(BN)_12_ contains a strained state, which probably increases the total energy. This is one reason why the relative energetic stability of Hex-(BN)_12_ with sp^2^ and sp^3^ hybridized bonds is slightly lower than c-BN, although sp^2^ hybridization is the energetically most favourable hybridization for B and N atoms.

**Table 1:** Crystal system (sys), space groups (groups), ratio of sp^2^ (*R*_sp^2^_) and sp^3^ (*R*_sp^3^_) B/N atoms in a unit cell, equilibrium density *ρ* (g cm^−3^), cohesive energy *E*_coh_ (eV/BN-pair), and energy band gap *E*_g_ (eV) for Hex-(BN)_12_ and several known BN structures (h-BN, dz2-BN, lz2-BN, 3D(6,0)-I, c-BN, bct-BN, w-BN, and yne-BN) at zero pressure

Stru.	Sys	Groups	*R*_sp^2^_	*R*_sp^3^_	*ρ* ours/ref.	*E*_coh_	*E*_g_ ours/ref.
h-BN	Hex	*P*6̄*m*2/187	100	0	2.27/2.270 (ref. 21)	17.33	4.68/4.778 (ref. 21)
dz2-BN	Orth	*Imm*2/44	100	0	2.547	16.87	3.33/3.42 (ref. 19)
lz2-BN	Orth	*Ima*2/46	80	20	1.917	17.09	2.04/1.53 (ref. 19)
Hex-(BN)_12_	Hex	*P*6_3_*cm*/185	50	50	2.722	17.02	3.25/4.42^a^
3D(6,0)-I	Tetra	*P*4_2_*mc*/105	33.33	66.67	3.34/3.21 (ref. 22)	16.81	3.34/3.21 (ref. 22)
c-BN	Cub	*F*4̄3*m*/216	0	100	3.47/3.48^b^	17.18	4.45/4.440 (ref. 21)
bct BN	Tetra	*P*4_2_/*mnm*/136	0	100	3.31/3.688 (ref. 21)	16.98	4.77/4.782 (ref. 21)
w-BN	Hex	*P*6_3_*mc*/186	0	100	3.46/3.49^b^	17.15	5.20/5.207 (ref. 21)
yne-BN	Hex	*P*6̄2*m*/189	—	—	—	16.26	4.21

a Reference HSE result.

b Reference experimental result.^48^

The dynamic stability of Hex-(BN)_12_ was also checked by calculating phonon dispersion along the high symmetry orientation in the Brillouin zone. We used linear response and finite displacement theories (shown in Fig. 3b) in the DFT framework to calculate the phonon spectrum in Hex-(BN)_12_. No imaginary frequencies were observed throughout the Brillouin zone, thus confirming the dynamic stability of this novel BN phase.

In addition, we calculated the elastic constants in order to test the mechanical stability of Hex-(BN)_12_. This phase has five independent elastic constants that correspond to its symmetric structure: *C*_11_, *C*_33_, *C*_44_, *C*_12_, and *C*_13_ are 498.084, 852.849, 222.766, 155.877, and 80.668 GPa, respectively. Obviously, these elastic constants satisfy the Born criteria for a hexagonal crystal:^49^*C*_44_ > 0, *C*_11_ >|*C*_12_|, (*C*_11_ + 2*C*_12_) × *C*_33_ > 2*C*_13_^2^. On the other hand, the mechanical stability confirms the plausibility of forming Hex-(BN)_12_.

The elastic constants of Hex-(BN)_12_ exhibit clear anisotropy. The elastic constant (*C*_33_) along the *Z*-direction (axial-direction) is slightly larger than the corresponding value in c-BN, while the elastic constants (*C*_11_ and *C*_22_) along the radial direction (*X*- and *Y*-directions) are about 35% smaller than that in c-BN. Nevertheless, the large magnitude of *C*_33_ indicates that the material has high resistance to linear compression along the axial direction.

In order to better understand the mechanical properties of this phase, Young's modulus was obtained along the three axes (Y_*x*_, Y_*y*_ and Y_*z*_), and the net Young's modulus (*E*) was estimate from the following equation:^44^*E* = 9*BG*/(3*B* + *G*)

The bulk modulus (*B*) and shear modulus (*G*) were then estimated from the elastic constants using Voigt–Reuss–Hill (VRH) approximations;^44^ these values are listed in Table 2. It is worth noting that Young's modulus along the *Z*-direction is comparable to the corresponding values from c-BN and w-BN, while Young's moduli along the *X*- and *Y*-directions are only about half the values in c-BN.

**Table 2:** Calculated Young's modulus along the three axes (Y_*x*_, Y_*y*_, and Y_*z*_), Young's modulus (*E*), bulk modulus (*B*), shear modulus (*G*), Pugh modulus (*k*), hardness (*H*_v_), and the behavior (Beh) for brittle (Bria) or ductility (Duc) of dz2-BN, lz2-BN, Hex-(BN)_12_, 3D(6,0)-I, c-BN, bct-BN, and w-BN at zero pressure. All parameters (except Pugh modulus and behavior) are in GPa

Stru.	dz2-BN	lz2-BN	Hex-(BN)_12_	3D(6,0)-I	c-BN	bct BN	w-BN	Ref.
Y_*x*_	699.68	687.13	445.64	570.28	719.25	711.97	910.27	Our work
Y_*y*_	81.28	90.48						Our work
Y_*z*_	443.59	50.61	832.95	768.36		934.74	1004.92	Our work
*E*	309.43	259.07	516.32	440.10	854.40	730.25	858.49	Our work
	326.60	246.40			856	749		19, 50 and 51
*B*	182.75	168.10	271.67	249.79	368.06	345.54	368.05	Our work
	212.40	175.50		258.40	376.19	348.35	375.24	19, 22 and 23
*G*	127.04	104.19	218.181	182.41	383.61	318.12	386.273	Our work
	131.30	97.30		156.10	381.52	309.44	384.17	19, 22 and 23
*k*	0.70	0.62	0.80	0.73	1.04	0.92	1.05	Our work
*H*_v_	19.24	14.32	33.13	26.11	65.18	49.85	66.01	Our work^a^
	62.40	49.14	59.59	53.39	64.25	61.13	63.62	Our work^b^
				56.10	46–80	58.77	50–60	22, 51 and 52
Beh	Bri	Bri	Bri	Bri	Bri	Bri	Bri	Our work

a Chen's result.

b Gao's result.

Modulus and hardness are often discussed together, even though the underlying deformations are fundamentally different.^45,53–55^ Here, considering the fact this phase is highly anisotropic and has a porous structure, we calculated the theoretical Vickers hardness (*H*_v_) using two different semi-empirical models. In the model of Chen *et al.*,^45^ the hardness is defined in terms of *G* and *B* using the following equation:*H*_v_ = 2(*k*^2^*G*)^0.585^ − 3where *k* = *G*/*B* is Pugh's modulus ratio.^44^ Chen's model returns a hardness of 33.13 GPa for Hex-(BN)_12_, which is about half the value for c-BN (66 GPa) but is close to the value for β-SiC and the latest proposed M-BN crystal (33.7–35.4 GPa).^20^ In the model of Gao *et al.*,^46^ the hardness is calculated using the following:*H*_v_ = 350 × *N*_e_^2/3^ × e^−1.191*f*_i_^/*d*^2.5^where *N*_e_ is the electron density of valence electrons per Å^3^ and *d* is the bond length (Å). Here, *d* is the average bond length. *f*_i_ is the Phillips ionicity, which was set to 0.256 for all B–N bonds.^20,22^ It has been widely used to estimate *H*_v_ in a number of crystals. Using this model, the value of *H*_v_ in this phase was estimated to be 59.59 GPa, which is comparable to the value for a c-BN crystal.

**Fig. 4:**
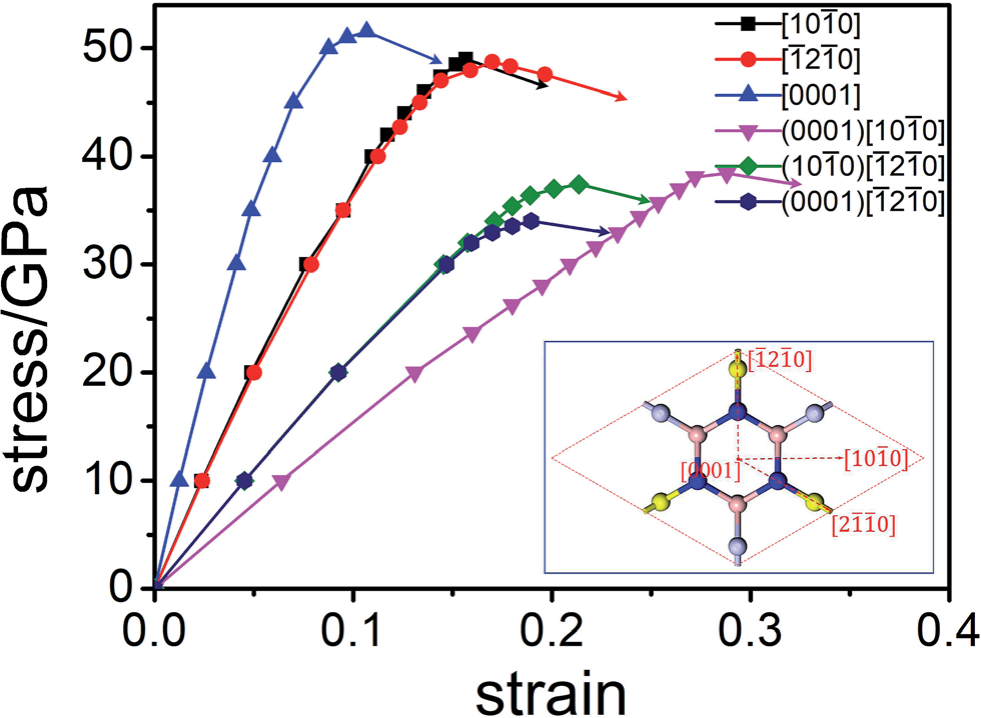
Orientation-dependent stress–strain relations for tensile and shear deformation in Hex-(BN)_12_.

The factors affecting total hardness are complex and include the type of elastic modulus, bond length, bond density, bond strength, and degree of covalent bonding. Remarkably, strength and hardness are often positively correlated. Ideal strength represents the maximum stress at which a perfect crystal becomes unstable and is more accurate for describing the mechanical strength than elastic constants.^56–58^ In this work, we evaluated the ideal tensile strengths of Hex-(BN)_12_ along the [101̄0], [1̄21̄0], and [0001] directions, as shown in Fig. 4. The corresponding tensile stresses of Hex-(BN)_12_ were found to be 49.00, 48.72, and 51.53 GPa with strains of 0.16, 0.17, and 0.11, respectively. Using the same strategy, the calculated ideal strength data for c-BN along the [111] direction was found to be 64.80 GPa, which agrees well with theoretical results from the literature^57,58^ and validates the computational methods used in this study. Clearly, Hex-(BN)_12_ has the lowest ideal tensile strength of 48.72 GPa at a strain of 0.17 along the [1̄21̄0] radial direction in the (0001) surface, similar to monolayer graphyne.^59,60^ The shear strength in the (0001)[101̄0], (101̄0)[1̄21̄0] and (0001)[1̄21̄0] slip systems are 38.44, 37.42, and 34.00 GPa at strains of 0.29, 0.21, and 0.19, respectively. The strength of the materials depends on the weakest strength component. All these results are consistent with the *H*_v_ values evaluated from Chen's semi-empirical model. We attribute the notable differences between the results from the two semi-empirical models to a large void network with large interstitial spacer. This fact results that the covalent bonds in the phase distributed ununiformly. Even though the sp^2^ and sp^3^ hybridized covalent bonds in this phase are strong, the large void reduces its mechanical properties. This is similar to previous research results on highly anisotropic, highly porous compounds.^61,62^ Therefore, we deduce that the hardness of Hex-(BN)_12_ should be approximately 33–40 GPa, which is close to the value for a β-SiC crystal, but the density of Hex-(BN)_12_ is lower than that of β-SiC by about 17%. Hard materials are those with hardness ranging from 20 to 40 GPa, while superhard materials are defined as materials with *H*_v_ exceeding 40 GPa.^63,64^ Both hardness values exceed 20 GPa, suggesting that Hex-(BN)_12_ at least is a hard material.

Table 2 shows that *E*, *B*, *G*, and *H*_v_ basically increase while the ductility decreases as the ratio of sp^3^-bonding atoms increases; lz2-BN and 3D(6,0)-I are exceptions. For instance, the ratio of sp^3^-hybridized atoms in orthorhombic lz2-BN is higher than in dz2-BN, but the corresponding *E*, *B*, *G*, *H*_v_, and brittleness values are lower than those in dz2-BN. This shows that the relative mechanical properties of BN materials are sensitive to hybridization and spatial structure. Nevertheless, sp^3^-hybridized B and N atoms are greater determinants of *E*, *B*, *G*, *H*_v_, and brittleness, while the ductility arises from the sp^2^-hybridized network. These mechanical values and density exhibit similar trends. Density often scales with mechanical properties,^65^ thus these density values are indicators of mechanical properties from another perspective. High density is consistent with superior mechanical properties,^65^ which again justifies its classification as a hard material.

**Fig. 5:**
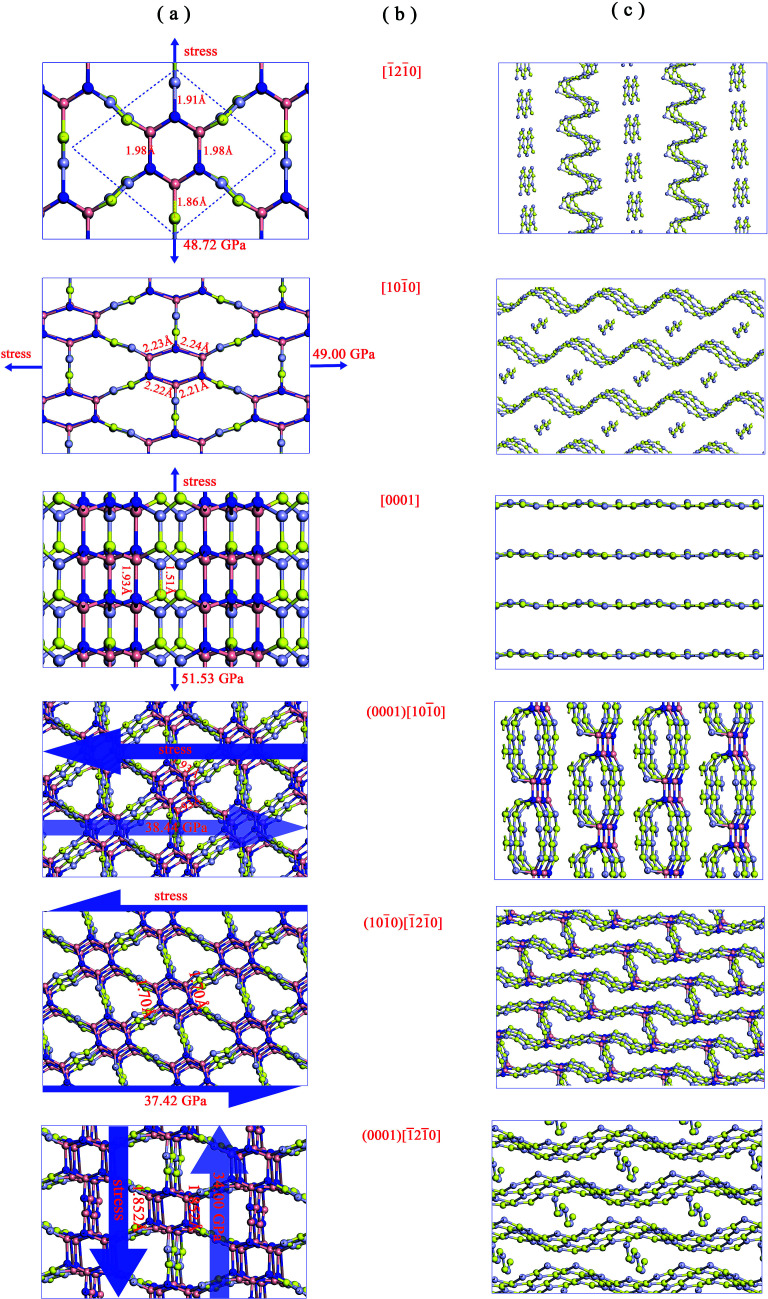
Critical and final states under different stress paths in Hex-(BN)_12_. Columns (a)–(c) show the critical state, stress paths (as labeled in Fig. 4), and final states when the stress exceed the critical tensile or shear force.

In order to know more about this behavior of sp^2^- and sp^3^-hybridized B and N atoms under stress, Fig. 5 shows the critical and final states under different stress paths for Hex-(BN)_12_. We can see that sp^3^–sp^3^ bonds in the BNNT component are more vulnerable to rupture than the sp^2^–sp^3^ bonds connecting BNNTs and BNNRs, and the sp^2^–sp^2^ bonds connecting BNNRs. This result agrees with the bond length results, where the sp^3^–sp^3^ bond lengths are longer than the sp^2^–sp^3^ bond lengths (by 0.07 Å) and sp^2^–sp^2^ bond lengths (by 0.17 Å). This is unlike the case of Hex-C_24_, where the sp^2^–sp^3^ bond is more vulnerable to rupture than sp^3^–sp^3^ and sp^2^–sp^2^ bonds, although it has a shorter bond length than the sp^3^–sp^3^ bond.^66^ From the critical state along the [1̄21̄0] direction, one can see that the sp^3^–sp^3^ bond ruptures first, followed by the sp^2^–sp^3^ bond. We can deduce that covalent bonds between sp^2^ and sp^3^-hybridized B/N atoms behave differently under stress, where the sp^3^–sp^3^ bond breaks first, followed by the sp^2^–sp^3^ bond and sp^2^–sp^2^ bond. This conclusion also can be supported by the ductility of the BN materials. c-BN, w-BN, and bct-BN are composed of tetrahedral sp^3^-hybridized B and N atoms, which cannot tolerate large deformations and are extremely brittle. Meanwhile, h-BN formed from sp^2^-hybridized atoms can sustain large distortions and is more ductile. These findings are similar to results from carbon. Therefore, Hex-(BN)_12_ consisting of sp^2^- and sp^3^-hybridized B/N atoms is quite interesting. Here, the Pugh modulus ratio (*k*) is used to separate brittle (above 0.57) and ductile (below 0.57) behavior.^67^ Our calculations show that *k* in Hex-(BN)_12_ is 0.80 (see Table 2), indicating that it is brittle. We attribute the brittleness of Hex-(BN)_12_ to sp^3^–sp^3^ covalent bonds. Moreover, the final states show that Hex-(BN)_12_ can be broken into BNNRs, BNNTs, BN nanosheets, and yne-BN due to tensile and shear forces.

**Fig. 6:**
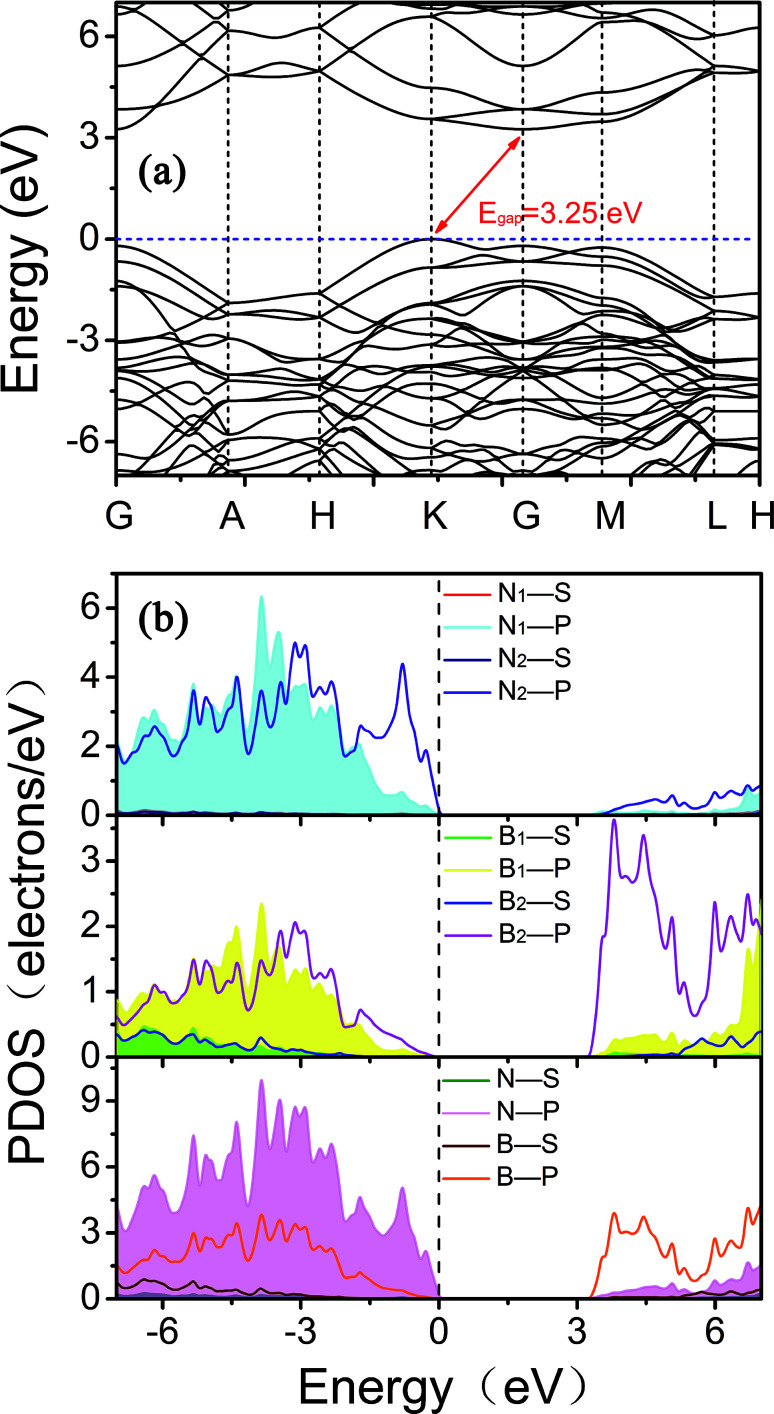
(a) Electronic band structures of Hex-(BN)_12_ along the high symmetry direction in the Brillouin zone. (b) Electron density of states (PDOS) projected on the s- and p-orbitals in B and N atoms with different hybridization as labeled in Fig. 1a. The energy at the Fermi level was set to zero. All results were obtained from DFT calculations with the PBE functional.

We then examined the electronic structure of Hex-(BN)_12_. The electronic band lines of Hex-(BN)_12_ obtained with the PBE functional are shown in Fig. 6a. Obviously, Hex-(BN)_12_ is a semiconductor with an indirect-band-gap of 3.21 eV at equilibrium. In light of the fact that the PBE functional always underestimates the band gap of semiconducting materials, we conducted more accurate DFT calculations with an HSE hybrid functional; the band gap was found to be approximately 4.42 eV.

**Fig. 7:**
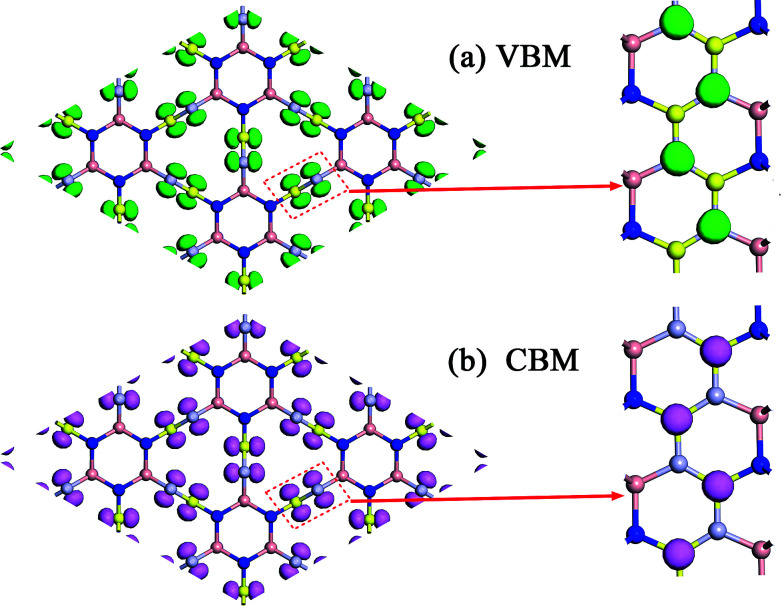
Isosurfaces of the Kohn–Sham wavefunctions of the (a) VBM and (b) CBM states in Hex-(BN)_12_. The isovalue was set to 0.05. Top and side views are plotted in the left and right columns.

To explain the origins of the electronic band structures, the electron density of states (PDOS) projected on the s- and p-orbitals in B and N atoms (as labelled in Fig. 1) are shown in Fig. 6b. Clearly, the electronic states in the region near the Fermi level originate primarily from the 2p orbitals in the sp^2^-hybridized B and N atoms in the BNNR component, whereas the contribution from the sp^3^-hybridized atoms is very small. These features can also be visualized using isosurfaces of the Kohn–Sham wavefunctions for the valence band maximum (VBM) and conduction band minimum (CBM), as shown in Fig. 7. The spatial distribution of the wavefunction implies that sp^2^-hybridized B and N atoms can gain electrons more easily than sp^3^-hybridized atoms in Hex-(BN)_12_. One can conclude that sp^2^ bonded atoms readily conduct electricity in BN materials with sp^2^ and sp^3^ hybridization based on results in the literature.^20,34^ This result agrees well with the effects of sp^2^- and sp^3^-hybridized B and N atoms on strength and ductility.

A number of results from experiments and theoretical calculations show that using precursors to seek new BN materials is feasible. Dai *et al.* predicted two types of porous BN networks that can be obtained with BNNRs.^19^ Xiong *et al.* and Hao *et al.* reported phase transitions of BNNT bundles under pressure.^22,68^ Inspired by the process of computing ideal strengths, we assumed two methods for synthesizing this new material based on the work of the aforementioned authors. First, Hex-(BN)_12_ can be obtained from direct compression of multilayer yne-BN. We then tentatively studied the structural transition of AB stacking in yne-BN to form Hex-(BN)_12_. The interlayer spaces are gradually compressed, and the yne-BN layers begin to buckle and bind together, finally converting into stable Hex-(BN)_12_. No transition state is found for this transition path. In addition, the transformation of yne-BN into Hex-(BN)_12_ is an exothermic process. The exothermic feature implies that fabrication of Hex-(BN)_12_ from yne-BN can be achieved under less rigorous conditions. Second, Hex-(BN)_12_ can be obtained from nanosheets, BNNTs, and BNNRs under pressure.^19,22,34,68,69^ Note that the major obstacle preventing new BN materials from being obtained is the limitation of a high-quality BN precursor. Today, successful preparation of BN nanosheets,^15,70^ single-walled BN nanotubes (SWBNNT),^71,72^ multi-walled BNNTs,^73,74^ and BNNRs^75,76^ provides fundamental building blocks for 3D BN polymorphs. A few new BN structures built using either BN nanosheets, nanotubes, or nanoribbons were reported in the literature.^19,22,34,68,69,77,78^ There is reason to believe that the special properties of Hex-(BN)_12_ will arouse further experimental efforts in producing this novel family of BN compounds.

## Conclusions

First-principles calculations were used to examine a new, hard, and semiconductive BN phase called Hex-(BN)_12_. The Hex-(BN)_12_ has equal numbers of sp^2−^ and sp^3−^ hybridized B and N atoms. Its energetic, dynamic, and mechanical stability was also unambiguously confirmed from calculations of the cohesive energy, phonon spectrum, and elastic constants. It can be obtained from multilayer yne-BN, nanosheets, BNNTs, and BNNRs under pressure. Hex-(BN)_12_ has unequal bond lengths and bond angles in these hybrid orbitals due to its structural characteristics, which reduce its relative energetic stability. The hardness of Hex-(BN)_12_ was estimated to range from 33 to 40 GPa. The calculated ideal tensile and shear strengths along highly symmetric directions are greater than 48.72 and 34.00 GPa, respectively. The covalent bonds between sp^2^- and sp^3^-hybridized B and N atoms behave differently under stress and can be broken into BNNRs, BNNTs, BN nanosheets, or yne-BN when tensile and shear forces are applied. The electronic states in the region near the Fermi level primarily arise from 2p orbitals in the sp^2^-hybridized atoms. In addition, we can deduce from the present and prior results that sp^3^-bonded B and N atoms guarantee higher mechanical properties, while sp^2^-bonded atoms ensure ductility and even conductivity in the BN family, although all these properties will change as the spatial structure changes. We expect that the results presented here will provide a new understanding of the structural, mechanical, and electronical properties of BN materials containing sp^2^- and sp^3^-hybridized B and N atoms.

## Conflicts of interest

There are no conflicts to declare.

## Supplementary Material
